# Supplementary material for: Bacterial Adhesion of TESPSA and Citric Acid on Different Titanium Surfaces Substrate Roughness: An In Vitro Study with a Multispecies Oral Biofilm Model
Source: Materials (Basel). 2023 Jun 25;16(13):4592. doi: 10.3390/ma16134592 (PMC10342536; doi:10.3390/ma16134592)
Supplement: Supplementary file 1 [file materials-16-04592-s001.zip › materials-2361579-supplementary.pdf]

Supplementary files

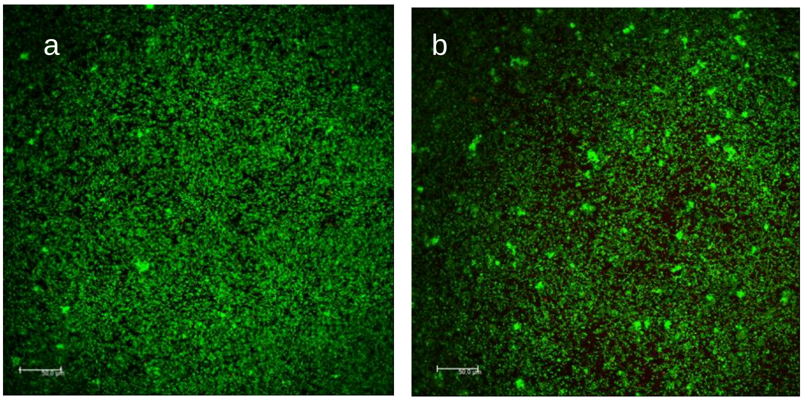

Figure S1. CLSM images from *L* surface at (a) 12 and (b) 24 h at 25x.

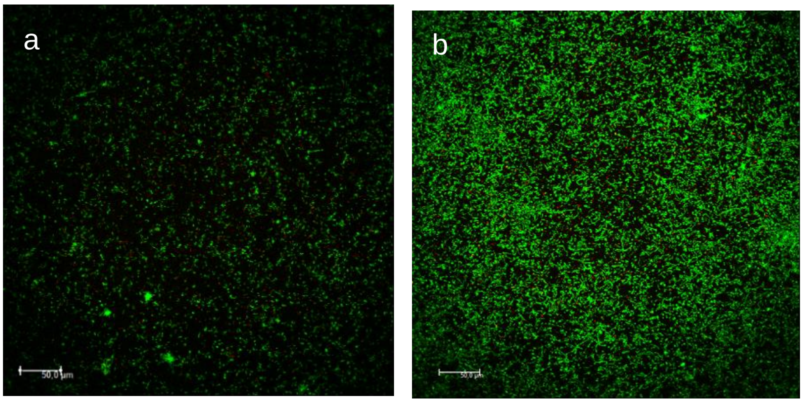

Figure S2. CLSM images from *L-TESPSA surface* at (a) 12 and (b) 24 h at 25x.

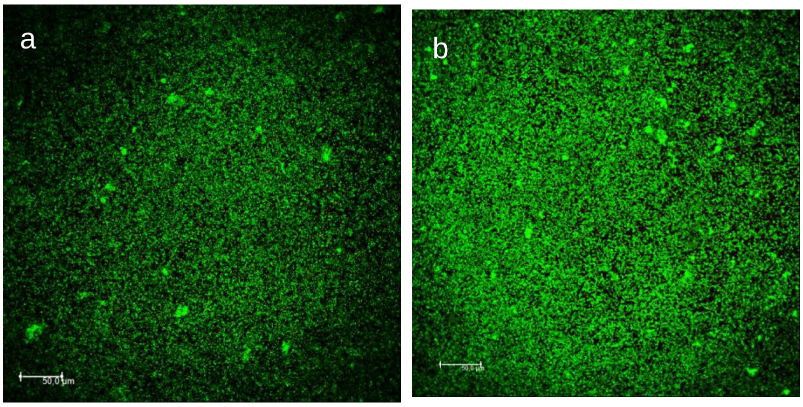

Figure S3. CLSM images from *M surface* at (a) 12 and (b) 24 h at 25x.

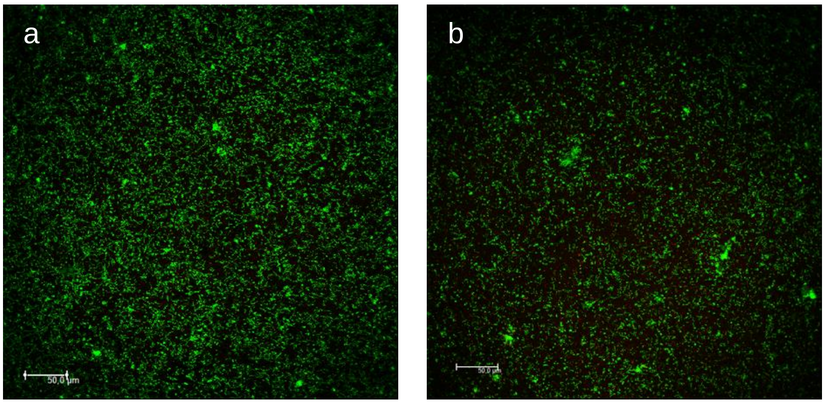

Figure S4. CLSM images from *M-TESPSA* surface at (a) 12 and (b) 24 h at 25x.

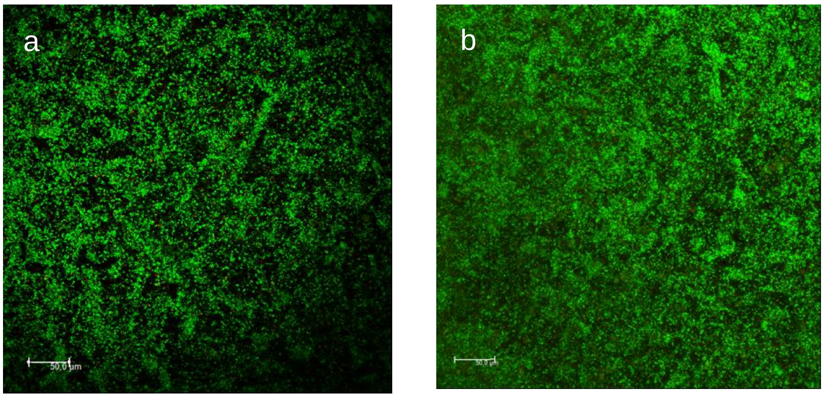

Figure S5. CLSM images from *H* surface at (a) 12 and (b) 24 h at 25x.

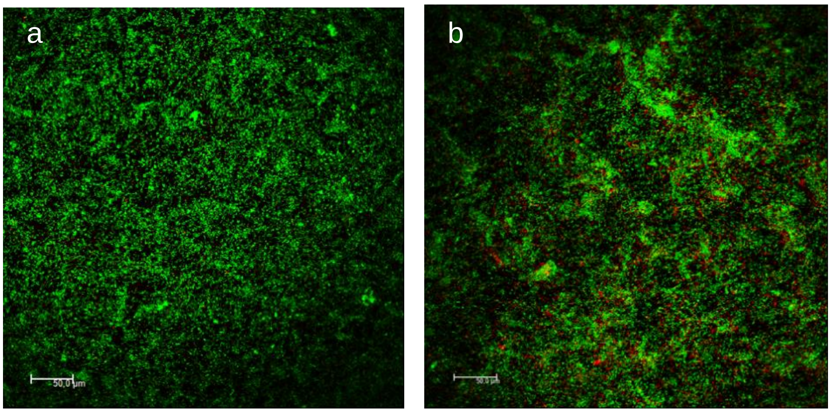

Figure S6. CLSM images from *H-TESPSA* surface at (a) 12 and (b) 24 h at 25x.

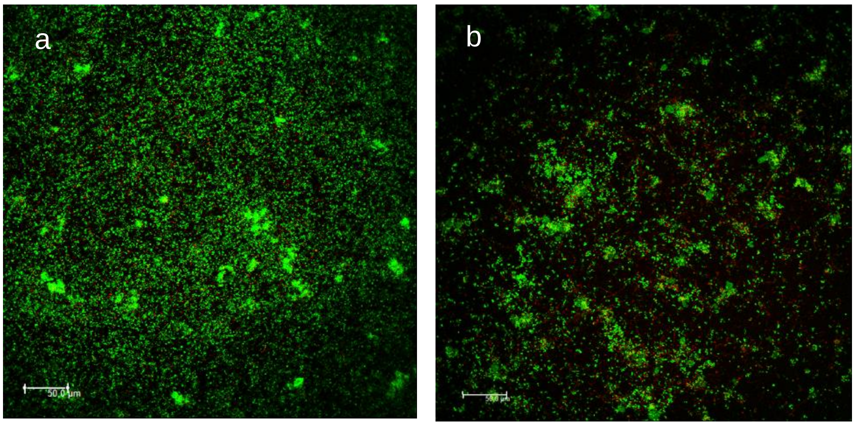

Figure S7. CLSM images from *CA* surface at (a) 12 and (b) 24 h at 25x.

Table S1. *P* values comparisons of the total bacterial load adhered to each surface measured by qPCR at 12 h.

Surface name	*L*	*L*-TESPSA	*M*	*M*-TESPSA	*H*	*H*-TESPSA
L-TESPSA	0.500					
M	0.248	0.217				
M-TESPSA	0.443	0.420	0.326			
H	0.487	0.458	0.304	0.515		
H-TESPSA	0.168	0.252	0.449	0.199	0.174	
CA	0.441	0.412	0.41	0.347	0.367	0.124

Table S2. *P* values comparisons of the total bacterial load adhered to each surface measured by qPCR at 24 h.

Surface name	*L*	*L*-TESPSA	*M*	*M*-TESPSA	*H*	*H*-TESPSA
L-TESPSA	0.475					
M	0.465	0.486				
M-TESPSA	0.319	0.266	0.257			
H	0.186	0.308	0.249	0.429		
H-TESPSA	0.437	0.436	0.447	0.194	0.391	
CA	0.193	0.219	0.196	0.441	0.491	0.239

Table S3. *P* values comparisons of the total bacterial load adhered among antibacterial surfaces measured by qPCR at 12 h.

Surface name	*L*-TESPSA	*M*-TESPSA	*H*-TESPSA
M-TESPSA	0.403		
H-TESPSA	0.062	0.072	
CA	0.308	0.279	0.021*

*p value <0.05.

Table S4. *P* values comparisons of the total bacterial load adhered among antibacterial surfaces measured by qPCR at 24 h.

Surface name	*L*-TESPSA	*M*-TESPSA	*H*-TESPSA
M-TESPSA	0.190		
H-TESPSA	0.357	0.131	
CA	0.141	0.357	0.124

Table S5. *P* values comparisons of the bacterial proliferation from 12 to 24 h measured by qPCR.

Surface name	*L*	*L*-TESPSA	*M*	*M*-TESPSA	*H*	*H*-TESPSA
L-TESPSA	0.348					
M	0.299	0.451				
M-TESPSA	0.233	0.106	0.075			
H	0.142	0.059	0.058	0.432		
H-TESPSA	0.234	0.412	0.437	0.053	0.056	
CA	0.096	0.046*	0.040*	0.348	0.425	0.054

*p value <0.05.

Table S6. *P* values comparisons of the bacterial mortality measured by qPCR at 12 h.

Surface name	*L*	*L*-TESPSA	*M*	*M*-TESPSA	*H*	*H*-TESPSA
L-TESPSA	0.474					
M	0.450	0.427				

M-TESPSA	0.621	0.508	0.437			
H	0.582	0.408	0.470	0.418		
H-TESPSA	0.425	0.483	0.430	0.437	0.425	
CA	0.546	0.398	0.580	0.451	0.420	0.429

Table S7. *P* values comparisons of the bacterial mortality measured by qPCR at 24 hours.

**Surface name**	***L***	***L*-TESPSA**	***M***	***M*-TESPSA**	***H***	***H*-TESPSA**
L-TESPSA	0.512					
M	0.418	0.469				
M-TESPSA	0.488	0.571	0.544			
H	0.645	0.828	0.594	0.488		
H-TESPSA	0.592	0.559	0.448	0.438	0.443	
CA	1.000	1.000	0.599	0.573	0.524	0.451

Table S8. *P* values comparisons of the total bacterial area measured by CLSM at 12 h.

**Surface name**	***L***	***L*-TESPSA**	***M***	***M*-TESPSA**	***H***	***H*-TESPSA**
L-TESPSA	0.506					
M	0.473	0.513				
M-TESPSA	0.472	0.400	0.367			
H	0.369	0.385	0.398	0.503		
H-TESPSA	0.350	0.484	0.841	0.500	0.483	
CA	0.388	0.608	1.000	0.469	0.469	0.488

Table S9. *P* values comparisons of the total bacterial area measured by CLSM at 24 h.

**Surface name**	***L***	***L*-TESPSA**	***M***	***M*-TESPSA**	***H***	***H*-TESPSA**
L-TESPSA	0.436					
M	0.370	0.409				
M-TESPSA	0.285	0.325	0.415			
H	0.277	0.303	0.365	0.413		
H-TESPSA	0.383	0.379	0.242	0.232	0.379	
CA	0.439	0.399	0.253	0.285	0.318	0.420

Table S10. *P* values comparisons of the live bacteria area measured by CLSM at 12 h.

**Surface name**	***L***	***L*-TESPSA**	***M***	***M*-TESPSA**	***H***	***H*-TESPSA**
L-TESPSA	0.464					
M	0.441	0.467				
M-TESPSA	0.484	0.431	0.383			
H	0.392	0.440	0.477	0.481		
H-TESPSA	0.402	0.428	0.778	0.465	0.451	
CA	0.379	0.551	1.000	0.483	0.461	0.470

Table S11. *P* values comparisons of the live bacteria area measured by CLSM at 24 h.

**Surface name**	***L***	***L*-TESPSA**	***M***	***M*-TESPSA**	***H***	***H*-TESPSA**
L-TESPSA	0.440					
M	0.357	0.392				
M-TESPSA	0.261	0.297	0.428			
H	0.299	0.263	0.367	0.424		
H-TESPSA	0.378	0.407	0.253	0.348	0.358	
CA	0.433	0.418	0.311	0.277	0.250	0.452

Table S12. *P* values comparisons of the dead bacteria area measured by CLSM at 12 h.

Surface name	*L*	*L*-TESPSA	*M*	*M*-TESPSA	*H*	*H*-TESPSA
L-TESPSA	0.183					
M	0.488	0.225				
M-TESPSA	0.112	0.012*	0.102			
H	0.256	0.043*	0.260	0.250		
H-TESPSA	0.135	0.022*	0.120	0.488	0.254	
CA	0.276	0.391	0.248	0.019*	0.090	0.023*

*p value <0.05.

Table S13. *P* values comparisons of the dead bacteria area measured by CLSM at 24 h.

Surface name	*L*	*L*-TESPSA	*M*	*M*-TESPSA	*H*	*H*-TESPSA
L-TESPSA	0.466					
M	0.391	0.419				
M-TESPSA	0.453	0.487	0.447			
H	0.127	0.123	0.124	0.141		
H-TESPSA	0.133	0.116	0.148	0.121	0.488	
CA	0.206	0.176	0.118	0.183	0.395	0.427

Table S14. *P* values comparisons of the total bacterial volume measured by CLSM at 12 h.

Surface name	*L*	*L*-TESPSA	*M*	*M*-TESPSA	*H*	*H*-TESPSA
L-TESPSA	0.408					
M	0.519	0.431				
M-TESPSA	1.000	0.439	0.586			
H	0.580	0.559	0.677	0.387		
H-TESPSA	0.842	0.565	1.000	0.405	0.497	
CA	0.469	0.552	0.519	0.393	0.479	0.529

Table S15. *P* values comparisons of the total bacterial volume measured by CLSM at 24 h.

Surface name	*L*	*L*-TESPSA	*M*	*M*-TESPSA	*H*	*H*-TESPSA
L-TESPSA	0.418					
M	0.365	0.400				
M-TESPSA	0.313	0.353	0.412			
H	0.200	0.210	0.273	0.262		
H-TESPSA	0.285	0.249	0.207	0.163	0.052	
CA	0.394	0.382	0.303	0.299	0.107	0.323

Table S16. *P* values comparisons of the live bacteria volume measured by CLSM at 12 h.

Surface name	*L*	*L*-TESPSA	*M*	*M*-TESPSA	*H*	*H*-TESPSA
L-TESPSA	0.425					
M	0.608	0.595				
M-TESPSA	0.757	0.467	1.000			
H	0.512	0.514	0.861	0.442		
H-TESPSA	0.463	0.530	0.704	0.436	0.490	
CA	0.524	0.601	1.000	0.416	0.578	0.560

Table S17. *P* values comparisons of the live bacteria volume measured by CLSM at 24 h.

Surface name	*L*	*L*-TESPSA	*M*	*M*-TESPSA	*H*	*H*-TESPSA
L-TESPSA	0.414					

M	0.367	0.406				
M-TESPSA	0.309	0.329	0.424			
H	0.141	0.193	0.262	0.303		
H-TESPSA	0.302	0.312	0.212	0.176	0.047*	
CA	0.412	0.390	0.316	0.297	0.121	0.348

*p value <0.05.

Table S18. *P* values comparisons of the dead bacteria volume measured by CLSM at 12 h.

**Surface name**	***L***	***L*-TESPSA**	***M***	***M*-TESPSA**	***H***	***H*-TESPSA**
L-TESPSA	0.274					
M	0.481	0.288				
M-TESPSA	0.027	0.005*	0.053			
H	0.234	0.072	0.260	0.131		
H-TESPSA	0.072	0.020*	0.091	0.298	0.299	
CA	0.287	0.480	0.269	0.007*	0.064	0.019*

*p value <0.05.

Table S19. *P* values comparisons of the dead bacteria volume measured by CLSM at 24 h.

**Surface name**	***L***	***L*-TESPSA**	***M***	***M*-TESPSA**	***H***	***H*-TESPSA**
L-TESPSA	0.493					
M	0.376	0.373				
M-TESPSA	0.531	0.513	0.352			
H	0.142	0.142	0.069	0.147		
H-TESPSA	0.088	0.064	0.054	0.116	0.358	
CA	0.207	0.203	0.112	0.209	0.415	0.232

Table S20. *P* values comparisons of biofilm mortality measured by CLSM at 12 h.

**Surface name**	***L***	***L*-TESPSA**	***M***	***M*-TESPSA**	***H***	***H*-TESPSA**
L-TESPSA	0.156					
M	0.395	0.230				
M-TESPSA	0.105	0.007*	0.073			
H	0.354	0.080	0.258	0.152		
H-TESPSA	0.149	0.012*	0.125	0.389	0.235	
CA	0.164	0.489	0.237	0.012*	0.090	0.015*

*p value <0.05.

Table S21. *P* values comparisons of biofilm mortality measured by CLSM at 24 h.

**Surface name**	***L***	***L*-TESPSA**	***M***	***M*-TESPSA**	***H***	***H*-TESPSA**
L-TESPSA	0.424					
M	0.471	0.441				
M-TESPSA	0.467	0.361	0.403			
H	0.093	0.111	0.076	0.119		
H-TESPSA	0.269	0.208	0.230	0.270	0.327	
CA	0.269	0.252	0.252	0.333	0.253	0.426
